# Precision cancer medicine and the doctor-patient relationship: a systematic review and narrative synthesis

**DOI:** 10.1186/s12911-023-02395-x

**Published:** 2023-12-14

**Authors:** Å. Grauman, M. Ancillotti, J. Veldwijk, D. Mascalzoni

**Affiliations:** 1 Centre for Research Ethics and Bioethics, Uppsala University, Box 564, Uppsala, SE-751 22 Sweden; 2 Erasmus School of Health Policy & Management, Erasmus University Rotterdam, Rotterdam, the Netherlands; 3 Erasmus Choice Modelling Centre, Erasmus University Rotterdam, Rotterdam, the Netherlands

**Keywords:** Precision medicine, Oncology, Cancer, Doctor-patient relation, Communication, Shared-decision making, Systematic review, Narrative synthesis

## Abstract

**Background:**

The implementation of precision medicine is likely to have a huge impact on clinical cancer care, while the doctor-patient relationship is a crucial aspect of cancer care that needs to be preserved. This systematic review aimed to map out perceptions and concerns regarding how the implementation of precision medicine will impact the doctor-patient relationship in cancer care so that threats against the doctor-patient relationship can be addressed.

**Methods:**

Electronic databases (Pubmed, Scopus, Web of Science, Social Science Premium Collection) were searched for articles published from January 2010 to December 2021, including qualitative, quantitative, and theoretical methods. Two reviewers completed title and abstract screening, full-text screening, and data extraction. Findings were summarized and explained using narrative synthesis.

**Results:**

Four themes were generated from the included articles (n = 35). *Providing information* addresses issues of information transmission and needs, and of complex concepts such as genetics and uncertainty. *Making decisions in a trustful relationship* addresses opacity issues, the role of trust, and and physicians’ attitude towards the role of precision medicine tools in decision-making. *Managing negative reactions of non-eligible patients* addresses patients’ unmet expectations of precision medicine. *Conflicting roles in the blurry line between clinic and research* addresses issues stemming from physicians’ double role as doctors and researchers.

**Conclusions:**

Many findings have previously been addressed in doctor-patient communication and clinical genetics. However, precision medicine adds complexity to these fields and further emphasizes the importance of clear communication on specific themes like the distinction between genomic and gene expression and patients’ expectations about access, eligibility, effectiveness, and side effects of targeted therapies.

**Supplementary Information:**

The online version contains supplementary material available at 10.1186/s12911-023-02395-x.

## Introduction

Precision medicine builds on the sub-classification of diseases using different features (e.g., genes, environment, and lifestyle) to tailor treatment and predict an individual’s disease risk and drug response. Thereby, treatment response can be optimized and serious, or even life-threatening, adverse events due to non-response can be avoided. The precision medicine approach combines different innovations and technologies; it can, for instance, involve algorithms, machine learning and artificial intelligence (AI), molecular profiling, next-generation sequencing (NGS), adaptive trials, and targeted treatments [1]. In many cases, precision medicine approaches are presented as a last treatment line, and targeted therapies can only be considered if a mutation is found. Therefore, only a selected group of patients will be eligible for precision medicine approaches. The term precision medicine is sometimes used synonymously with personalized medicine or stratified medicine [2]. However, the term personalized medicine emphasizes patient participation and preferences [3], and some have related it to patient-centeredness and holistic wellness [4]. Precision medicine does not, per se, include these concepts.

The progress and implementation of precision medicine have come furthest in oncology, likely because cancer is a genetic and molecular disease. Classifying various cancers based on their molecular origin enables the development of targeted therapies, prediction of drug response and toxicity, and prevention through the identification of carriers of mutations [5, 6]. Precision medicine often applies an experimental approach, where individualized research data is incorporated into clinical therapy treatment. It changes how clinical trials are usually conducted, for instance, in the number of research subjects, as very few or even only one patient participates [7]. Precision medicine is therefore surrounded by enhanced scientific uncertainties, and has blurred the line between therapy and research. Uncertainty in cancer treatment is not unique to precision medicine, but is also an acknowledged communication challenge in standard treatment [8]. A molecular tumor board (MTB) is a multidisciplinary team that can support oncologist treatment decisions in precision medicine. Based on genetic analysis and additional factors of the patient, the MTB assesses eligibility for targeted therapies and provides treatment recommendations [9].

The scientific uncertainties, high level of computation, and use of algorithms affect clinical decision-making; it may also affect the interaction between physician and patient. The severity of cancer and the vulnerability that cancer patients experience can increase patients’ trust in and dependence on their physician [10], making the doctor-patient relationship especially important in cancer treatment.

The doctor-patient relationship has been conceptualized and defined in various ways. One definition is “a consensual relationship in which the patient knowingly seeks the physician’s assistance and in which the physician knowingly accepts the person as a patient” [11]. Aspects defining a good doctor-patient relationship involve high levels of trust in doctors’ competence [12, 13], interpersonal openness [12, 13], mutual respect and knowledge of each other, and feelings of comfort and liking [12]. The doctor-patient relationship has also been conceptualized based on aspects of the psychotherapeutic alliance, where doctors’ affective behaviors are especially important for patient satisfaction. Such behaviors include being caring [12], helpful [13], honest [12], attentive, the ability to listen, communicate concerns, warmth [14], and showing empathic understanding [13]. In a poor doctor-patient relationship, patients feel unheard, disrespected, and out of partnership with their physicians [11]. Emanuel and Emanuel describe four models of doctor-patient relationship: the paternalistic model, the interpretive model, the deliberative model, and the informative model [15]. These models describe different perspectives on the goals of the physician–patient interaction, the physicians’ obligations, the role of patients’ values, and the conception of patient autonomy. The authors claim that the ideal physician–patient relationship is the deliberative model, which supports patient autonomy and requires that patients assess their own values and preferences [15].

Communication is a cornerstone of a good doctor-patient relationship. High-quality doctor-patient communication ensures a patient is included in the decision-making process, for instance, by providing them with information and asking about their illness perception [11]. Shared decision‐making (SDM) is a strategy where clinicians and patients jointly participate in making a health decision, having discussed the options and their benefits and harms, as well as having considered the patient’s values, preferences, and circumstances [16]. It respects patient autonomy and is especially appropriate under uncertainty and situations where there is no superior treatment alternative, which is frequently encountered in precision oncology [17], or when a patient decides or doctor advices not to take a treatment. Shared-decision making shows high agreement with the deliberative doctor-patient relationship model. For SDM to succeed, it is crucial that the patient is willing and able to actively engage in the information exchange [8].

The implementation of precision medicine and AI-based decision support systems is likely to have a huge impact on clinical cancer care, while the doctor-patient relationship is a crucial aspect of cancer care that needs to be preserved. This systematic review aims to map out perceptions and concerns regarding how the implementation of precision medicine will influence the doctor-patient relationship in cancer care so that threats against the doctor-patient relationship can be addressed.

## Methods

This systematic review was conducted according to PRISMA guidelines [18]. Both empirical and theoretical articles were included to capture different and complementary perspectives on the topic.

### Eligibility criteria

The eligibility criteria (Table 1) were designed according to the SPIDER criteria (sample, phenomena of interest, design, evaluation, research type), which are suitable for qualitative and mixed method research [19].

**Table 1:** Eligibility criteria according to SPIDER

Sample:	Cancer patients, health professionals, the general public, researchers, experts ≥ 18 years of age
Phenomenon of Interest:	Precision medicine in relation to the Doctor-Patient relationship in oncology
Design:	Case studies, interviews, surveys, editorials
Evaluation:	Perceptions and experiences (concerns, attitudes, expectations, hopes)
Research type:	Qualitative, quantitative, mixed methods, theoretical, opinion pieces

### Search strategy

A search strategy was developed with an academic librarian at Uppsala University library. Medline (via PubMed), SCOPUS, Web of Science, and Social Science Premium Collection were searched for papers published from January 2010 to December 2021. The time restriction was set to capture research on recent discoveries and clinical practice and not outdated speculative theories. The inclusion criteria were peer-reviewed papers written in English, relating to precision medicine or AI and doctor-patient relationship, oncology, and adult patients. Exclusion criteria were articles unrelated to the research question, pediatric oncology, systematic reviews, grey literature, conference protocols, and abstracts. The search strategy and documentation are presented in Supplementary file A.

### Identification of studies and data extraction

The articles identified in the search were uploaded to Rayyan [20]. ÅG and MA conducted a blinded double-screen of the title and abstract of all articles (stratifying papers into: inclusion, doubt, exclusion). Disagreements and doubtful cases were discussed until consensus was reached after removing the blinding. Full-text reading was completed of the included articles, divided by ÅG and MA. JV read a random selection of 10 of these papers to ensure agreement and avoid reviewer bias. Disagreements were discussed until consensus was reached. Data were extracted by ÅG and MA into an Excel sheet with pre-defined categories: title, author, year of publication, aim of the study, design, data collection, participants, sample size, location of the study, cancer type, precision medicine area, and conclusions of the study. It also included categories related to the doctor-patient relationship, e.g., trust (in doctor and tool), role, obligations, communication, interaction, autonomy, and shared decision-making. There was also the possibility to add information into an “other” column.

### Narrative synthesis

In this systematic review, we included articles that used different methods, looking for different aspects of how stakeholders perceive the implications of precision medicine on the doctor-patient relationship rather than evidence confirming these perceptions. The final selection of articles was included in a narrative synthesis, using text to summarize and explain the findings from the systematic search [21]. This method is particularly useful when linking together studies on different topics for reinterpretation [22]. The data were pooled together by findings related to the same category, regardless of the design of the study. The text was then summarized, condensed, and synthesized to provide a meaningful narrative that answered the research question.

### Quality assessment

The Mixed Methods Appraisal Tool (MMAT) [23] was selected to evaluate the methodological quality of the included studies. The MMAT establishes validity and reliability for summarizing overall quality across a range of study designs, thus ensuring a consistent approach. Quality appraisal was conducted by ÅG, and MA and JV independently evaluated a random sample of four papers each. All papers were included in this review regardless of quality ranking.

## Results

### Included studies

**Fig. 1:**
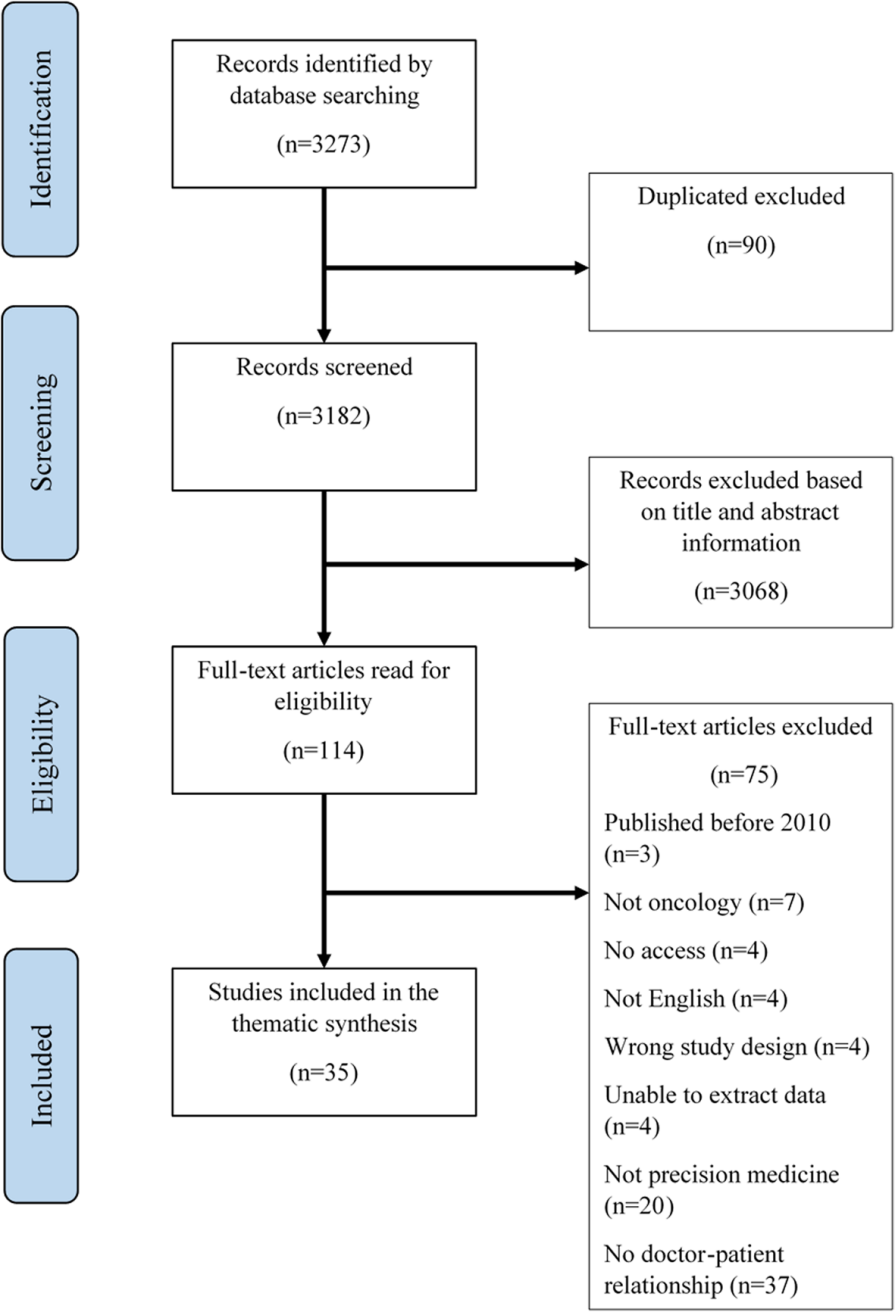
PRISMA (Preferred Reporting Items for Systematic Reviews and Meta-Analyses) flow diagram

The database search resulted in 3273 records. After the title-abstract screening, 114 articles remained and were read in full-text for eligibility (Fig. 1).

Thirty-five articles were included in the thematic synthesis (Table 2). It is noteworthy that the studies described in these articles were conducted (and the affiliation of the first author of the theoretical studies) in North America and Europe, with only two exceptions from China and Australia. The USA was overrepresented (*n* = 13), followed by the United Kingdom (*n* = 4), and Germany (*n* = 4). Most studies had a qualitative design (*n* = 20), one used mixed methods, three used cross-sectional surveys, and the remaining eleven were theoretical papers (comments, editorial, opinion pieces, etc.). Patients were the most common study population. Eight articles addressed precision medicine in general, while a substantial part of the articles addressed genomic testing and gene expression testing. Six articles focused on AI systems in health care.

**Table 2:** Summary of included studies

Qualitative studies					
**Author (year)**	**Country**	**Main topic**	**Design/method**	**Participants (n)**	**Cancer field**
Abe (2017) [24]	USA	Genomics-informed treatment decisions for radiation therapy	Semi-structured stakeholder meetings	Patients, surgeons, oncologists, industry representatives, biomedical informatics. *N* = 22	Prostate cancer
Bijlsma (2018) [25]	The Netherlands	Unsolicited genetic information obtained using NGS	Individual interviews	Patients *n* = 24	Cancer
Bombard (2014) [26]	Canada	Gene expression profiling	Individual interviews, focus group discussion	Oncologists, *n* = 14 patients, n = 28	Breast cancer
Bombard (2015) [27]	Canada	Gene expression profiling	Individuals interviews	Oncologists *n* = 14	Breast cancer
Costa (2021) [28]	Canada	Prognostic-based genomic testing	Focus group discussions	Patients, *n* = 26	Lymphoid Cancers
Dodson (2017) [29]	USA	Pharmacogenomics	Open-ended survey	Oncology nurses, *n* = 28	Cancer
Hamilton (2021) [30]	USA	Precision oncology	Focus group discussions	Oncology clinicians, *n* = 68	Breast, melanoma and thoracic cancer
Hamilton (2017) [31]	USA	Secondary germline findings from tumor genomic profiling	Individual interviews	Patients with advanced cancer, *n* = 40	Breast, bladder, colorectal, or lung cancer
Harris (2013) [32]	USA	Kirsten ras tumor mutation testing (KRAS)	Individual interviews	Oncologists, *n* = 34	Colorectal cancer
Kerr (2019) [33]	UK	Molecular cancer diagnosis/treatment	Individual interviews	Practitioners *n* = 25	Cancer
Mamzer (2017)^a^ [34]	France	Translational oncology research	Case study	Patient and expert committee members, *n* = not specified	Cancer
McCradden (2020) [35]	Canada	AI in health care research	Individual interviews	Patients, caregivers, and health-care providers, *n* = 30	Meningioma
Perry (2017) [36]	Germany	"Personalized" treatment research (e.g. biomarkers for stratification)	Individual interviews	Patients, *n* = 40	Colorectal cancer
Pellegrini (2011) [37]	France	Tumor gene expression analysis	Individual interviews	Patients, *n* = 37	Breast cancer
Pichler (2020) [38]	Germany	Molecular diagnosis	Individual interviews	Patients, *n* = 30	Cancer, advanced stage
Rattay (2018) [39]	UK	Predictive radiogenomics testing for breast radiation toxicity	Individual interviews	Patients, *n* = 21	Breast cancer
Rohrmoser (2019) [40]	Germany	Molecular diagnostics	Individual interviews	Patients, *n* = 30	Cancer, advanced stage
Steltzer (2020) [41]	Austria	Personalized medicine	Individual interviews	Patients *n* = 2 Health care professionals, *n* = 14	Cancer
Therond (2020) [42]	UK	BRCA genetic testing	Observation and individual interviews	Patients, *n* = 25	Breast, ovarian
Wright (2019) [43]	UK	BRCA 1 & 2 testing	Individual interviews and team meetings	Clinicians *n* = 19, teams	Breast cancer
**Mixed-methods**					
Best (2020) [44]	Australia	Somatic molecular profiling (MP) test results	Survey and individual interviews	Patients survey *n* = 1299, interview *n* = 20	Advanced cancers
**Quantitative methods**					
Issa (2013) [45]	USA	Novel personalized medicine genomic diagnostics	Discrete Choice Experiment survey	Patients, *n* = 300	breast and colorectal
Soellner (2021) [46]	USA	AI for diagnostics	Survey	Public, *n* = 452	Skin cancer
Yang (2019) [47]	China	AI in medicine	Survey	Patients, *n* = 527	Cancer
**Theoretical, opinion pieces**					
Aminololama-Shakeri (2019) [48]	USA	AI in breast imaging radiology	Opinion paper		Breast
Ansmann (2018) [49]	Germany	Precision medicine	Commentary		Cancer
Bunnik (2021) [50]	The Netherlands	Genomic sequencing	Perspective		Cancer
Carter (2020) [51]	Australia	AI systems	Theoretical review		Breast cancer
Korngiebel (2017) [52]	USA	Pharmacogenomic tests and tests for inherited cancer risk	Review/theoretical		Cancer
Marchiano (2018) [53]	USA	Precision oncology	Communication		Cancer
McFarland (2017) [54]	USA	Precision medicine oncology and targeted therapies	Editorial		Cancer
McFarland (2017) [55]	USA	Precision medicine oncology	Editorial		Cancer
McGrath (2021) [56]	USA	Genetic and genomic testing	Perspective		Cancer
Stoeklé (2018) [57]	France	Precision medicine, Molecular tumor board (MTB)	Opinion paper		Cancer
Triberti (2020) [58]	Italy	AI health decision making	Perspective		Cancer

^a^The study by Mamzer et al. (2017) is a description of the involvement of patient representatives and to establish a long-term partnership integrating patient’s expectations. No formal qualitative analysis was performed. It was, therefore, not included in the quality assessment

### Quality assessment

Most studies were considered to be of sufficient quality. The most common quality issue was insufficient description of the methods, making it difficult to determine how the study was conducted and analyzed. Lack of quotes and unsuitable sampling strategies are further examples of quality issues. The full quality assessment of the empirical studies can be found in Supplement B.

### Providing information

The concerns about the impact of precision medicine on the doctor-patient relationship were not new or specific to precision medicine per se. Rather, precision medicine was perceived to bring further complexity to the challenges already encountered in communication and shared-decision making [41].

### Providing clear information while avoiding information overload

The provision of understandable information is crucial since it can protect patient autonomy, decrease patient distress, and enhance shared decision-making [49, 55]. Precision medicine involves increased amount of information due to the additional tests and treatment options, which can lead to information overload for the patients [24, 39, 55]. Meanwhile, patients express that they lack information about precision cancer medicine [26, 38]. PM also includes new and complex concepts, e.g., targeted therapies, genetics, and algorithms. Oncologists may lack training in how to communicate such concepts, while many patients have never heard of these terms, which can create a barrier to communication [29, 48, 50]. Physicians experience that patients’ understanding of PM differs from their own [30] and express doubt that patients understand the given information [27]. Furthermore, oncologists have shown to differ in their communication and framing of concepts within precision medicine [27, 32], why standardization of information to improve the provision of information has been requested [29, 38].

### Providing and receiving information about genetics

Precision medicine includes genetic testing to guide therapy selection [29]. While gene expression profiling is restricted to the tumor, genomic profiling has far-reaching consequences. Besides the answer to a clinical question, genomic testing can produce three other types of results: suspected germline mutations, variants of uncertain significance (VUS), and unsolicited findings relating to other conditions. Such genomic findings may have clinical, psychological, and social impacts on the patients and their family members [29, 41, 50, 55]. The increased use of genomic testing comes with new tasks for health professionals, such as helping patients interpret test results, offering psycho-oncological support and genetic counseling [25, 29, 38], and coordinate further testing within the family [25, 29]. Health care professionals are ethically and sometimes legally required to inform patients of the purpose of genetic tests, the risks and uncertainties, and the implications of its results, including unexpected results, as part of the informed consent process [43, 50, 52]. The provision of information can help patients to prepare for eventual clinical and psychosocial consequences of genetic findings, and gives the opportunity to autonomously decide whether or not to receive different types of genomic information [50].

Patients’ preferences for receiving different genetic information differ [28, 29, 39, 40]. Physicians, therefore, need to explore how much information the individual patient wants [29, 33, 56]. Differences in preferences can relate to whether the results are actionable, i.e., if the results will inform cancer prevention or treatment [28, 44], or whether there is an available treatment for symptom management [39]. Gathering non-actionable information can constitute a vital coping mechanism to reduce distress in a threatening situation for some patients [40]. Likewise, test results may provide patients with accurate expectations about the course of their treatment, and help them know what to expect [39]. On the other hand, patients may decline both genetic testing and genetic information due to concerns about receiving potentially hereditarily relevant information [40], or not feeling equipped to interpret the findings [28, 44].

Although being very different, patients can misunderstand the terms ‘genomic’ and ‘gene expression’ for their constitutional genes, which can lead to the fear that cancer can be transmitted to offspring’ [37]. The mix-up of the concepts may also influence the attitude toward privacy, where the individual patient’s germline genetic is considered more sensitive than tumor genomics [24]. This makes communication revolving around genomic testing and potentially genetic profiling a sensitive matter.

### Communicating uncertainty

Precision medicine involves uncertainty due to a lack of evidence. To maintain patients’ trust and promote autonomy, it is important to be explicit about uncertainty and gaps in knowledge [42, 55]. However, clinicians express that uncertainty is problematic and that it can be perceived as frightening to both clinicians and patients [33]. Furthermore, oncologists fear that discussing uncertainty with patients may make the patients think that they are not competent [30]. Difficulties with discussing therapeutic uncertainty may result in physicians not disclosing all of the information, not talking about it, or oversimplifying the information, which may risk misleading the patients [55]. A trusting relationship between provider and patient can be reassuring to withstand uncertainty [38, 55]. Furthermore, by communicating uncertainties in advance of testing, patients can tolerate the uncertainty and be willing to receive uncertain test results [28].

### Making decisions in a trusting relationship

Precision medicine involves decisions about, among other things, genetic and molecular testing, receiving test results, multiple treatment options, and participation in clinical trials [34, 38, 49, 52]. The broad use of health data in PM increases the risk to personal privacy by data leakage and re-identification [35, 51]. Patients, therefore, also need to make informed decisions about the use and re-use of data [34]. A lot of the technologies in PM are costly. To avoid overuse of tests, e.g., radiogenomics tests, the physician needs to determine patients’ willingness to act on the results in advance of ordering tests, hence the patient’s decision to have a mastectomy [27, 39].

Many patients feel a strong trust in their doctors, which, in turn, makes them perceive the doctor as a crucial partner in decision-making [47]. Patients may therefore request their physicians’ opinion on what to decide, on the basis that patients think their doctors will act in their best interests [28, 31, 39, 45]. It could, for instance, involve trusting the physician to sort information from test results and only tell them what is of importance, thus avoiding unhelpful results [44]. Furthermore, patients may deal with anxiety and fear at the decision moment [24] and be unwilling or unable to participate in the decision-making process, and thereby handing over the decision making to the physician [41, 50].

Algorithms and AI technology support decision-making tools and prediction models in PM. Traditionally, when physicians make decisions without algorithms, they are generally able to provide some explanation about the grounds of the decisions. However, decisions about diagnostic, prognostic, and treatment made by the algorithms may lack transparency in how the decisions are made (the so-called ‘black box’ problem). This obscures the ability to explain the decisions to a patient [51, 58]. Furthermore, some physicians feel that decisions need to be made collectively and require conversations with patients and their families, which can make the physician reluctant to delegate decisions entirely to machines [35]. Some physicians reject the idea of allocating patients to treatment based on an algorithm predicting their probability of benefiting, stating that trying is important and that all patients deserve a chance. The unwillingness among physicians to base their decisions on outcomes of prediction models may be reinforced by perceptions of the uncertainty of the prediction, namely that not all relevant factors for the evaluation of the individual may have been included [35]. That could also make physicians not take a certain test since it enables them to continue treatment anyway [30, 33]. Likewise, many patients perceive doctors as irreplaceable and better suited than an AI to deal with complicated situations [47]. The lack of transparency about how decisions are made when using AI systems may influence patients’ trust in clinicians and healthcare institutions and may cause a shift in attributions of responsibility for the decision [51, 58]. The use of AI, as well as administrative tasks connected to precision medicine, such as coordinating tests and determining patient clinical trial eligibility is expected to decrease human interaction and therefore have a negative impact on the doctor-patient relationship [26, 30, 54]. On the contrary, others think that introducing AI in health care will positively impact the doctor-patient relationship, e.g., free time from administrative tasks. Health professionals may spend this time on interaction and communication with patients, empathic listening, thus improve shared decision-making [48, 51, 58].

### Managing negative reactions of non-eligible patients

Precision medicine gives patients hope for a prolonged life [28, 40]. Some patients perceive gene expression profiling tests to be special, because of its barriers to access and since it is not included in standard treatment [26]. Patients have also expressed that molecular diagnostics made them feel special since that implied more attention [40]. However, patients’ expectations about precision medicine can be too optimistic, e.g., thinking that all new treatments will be effective for them [28, 30], or expecting tailored treatments [30]. Media coverage and direct-to-consumer marketing can contribute to patients’ optimistic expectations and make patients ask for specific tests and targeted approaches. At the same time, this can increase the pressure oncologists feel to offer the test [26, 30, 41, 55]. Physicians state that patients may experience disappointment and anger when they try to readjust these expectations [30, 55]. These exaggerated expectations may also constitute a barrier to facilitating a critical discussion [28].

Precision medicine moves away from standard treatments given to all patients towards personalized treatment selection, thereby avoiding unnecessary treatment and the risk of serious side-effects [27, 29, 52]. However, treating patients differently may affect the interaction between patient and doctor. For instance, some patients may view access to tests as a right [26], and physicians experience that patients are likely to feel that they are unfairly denied access when they do not qualify for tests [26]. Physicians have also been shown to decide differently about who should have molecular testing [26]. Therefore, the feelings and concerns about unequal access may, in some cases, be justified.

When a test shows a low probability that the treatment will be effective, or the patient is not eligible for targeted treatments, and there is no other treatment to offer, precision medicine may be perceived as narrowing down treatment options for the patient [29]. That situation brings an “unwelcomed certainty” to the patient’s situation [30, 33]. Explaining to patients that they are not eligible for treatments can be difficult and make patients feel abandoned and lose hope [34].

### Conflicting roles in the blurry line between clinic and research

Clinical trials may be considered as treatment options within the precision medicine context because they provide some patients with a last chance of recovery [44, 57], creating a blurry line between clinical and research spheres [53]. The blurry line might create conflicting roles for doctors acting not only as physicians but also as researchers, recruiting patients into clinical trials for the sake of research goals [36], knowing that the individual study participants are unlikely to benefit [41]. After all, clinical trials often aim at obtaining data to advance cancer therapeutics for future patients [53, 57].

Patients’ optimism and unawareness of the small chances of an actual personal benefit can make them consent to precision medicine trials with the wrong expectations [36, 38, 53]. It is, therefore, crucial that oncologists openly discuss hopes and expectations as well as actual chances of a benefit from the exploratory nature of PM [38]. However, patients’ perceived benefit from participating in research can involve other aspects than cure, like more intense supervision [36], willingness to please the physician [35, 36], and an opportunity to feel meaningful, especially among participants who hold little hope for their own recovery [40, 44].

## Discussion

The narrative synthesis of this systematic review describes that precision medicine can influence the doctor-patient relationship in various ways. The major themes are related to communication, genetic information, trust, informed consent, shared decision-making, algorithms and AI, and the blurry line between care and research. These themes are not unique to precision medicine, but there is a higher complexity connected to precision medicine compared to other areas of medicine [41, 59].

One issue addressed in this review was the blurry line between research and clinic. This relates to the well-debated phenomenon called the “therapeutic misconception” that often but not always also involves overestimation of clinical benefit and underestimation of the risk of harm involved in a clinical trial [60]. Hansson & Hakama state that it is an ethical problem when patients are given the impression that the overall goal of a clinical trial is therapy [61], but argue that the main problem is not the patient misconception, but rather the role of the doctor. Since the responsibility to understand basic principles of science should be assumed for the doctor, but not for the patient, they suggest that the doctor has a special moral responsibility. They suggest that the attending doctor should not recruit their own patients into trials as a solution to the problem. Instead, the doctor should hand over the task of informing and all decisions regarding participation in the trial to an independent representative of the trial [61]. However, based on other findings of this study, it is likely that in precision medicine contexts the patient will still turn to their attending doctor for guidance before making a decision.

To benefit from precision medicine, it is crucial that physicians trust and act on the outcomes of decision tools. A study from the US found that physicians often made decisions in collaboration with their patients. However, these decisions often deviated from the recommendations from MTB, which was seen as a sign of physician preferences to choose established therapies [62]. There were examples in our results where physicians disregarded outcomes of decision tools involving low probabilities of a treatment benefiting the patient, with the argument that everybody deserves a chance of being cured and the presence of uncertainty in the model. Likewise, in a study by Heßling & Schicktanz, German physicians stated that the most important thing is that no patient is deprived of a treatment [63]. They would, therefore, rather accept a test with a low positive predictive value and risk that non-responders would be falsely classified as responders than accept a low negative predictive value. Hence, they chose to meet uncertainty with the risk of overprescribing therapy [63]. This may imply that physicians do not trust the decision models enough, which may lead to exposing patients to unnecessary side effects [63], and pose a threat to the scarce resources countries have in health care. It may also imply that physicians think that it goes against their moral obligation to do everything in their power to help their patients. Expecting that therapies may be refused to them is a reason patients may fear being classified as non-responders [63]. In a study by Sinding et al. (2010), patients were just like the doctors willing to accept a treatment despite the low probability of being beneficial. The patients expressed that it made them feel that they had tried everything, which made it easier to cope mentally with an eventual recurrence [64]. Furthermore, the patients did not find it sufficient to make decisions on their own based on statistics about risks and benefits. Instead, they requested physicians gut feelings and wanted their opinion on what to do. In contrast, the physicians seemed reluctant to share it, leaving the responsibility for the decision entirely to the patient. Sinding et al. (2010) suggested that accepting the treatment with low probabilities removed the patients’ perceived responsibility for an eventual recurrence. The study also found a belief that there are right and wrong treatment decisions, and that recurrence is the result of wrong decisions, while cancer treatment does not, in fact, guarantee a cure [64]. It may therefore be more useful to try to define what a wrong decision is and discuss what thresholds for treatment effectiveness probability should be accepted. Willingness to accept low probabilities must also be seen in the context of other available treatment options, and whether the treatment is the only option left. In this example from Sinding et al. (2010), the decision-making process passes from a shared decision-making deliberation process to a situation where the physician does not offer assistance, leaving the patient abandoned with her decision. However, the physician cannot renounce the responsibility of decision making, while the patient can, since patient autonomy is a right and not an obligation.

Much of the results emphasize the need to provide patients with information to enable informed decisions about, e.g., genetic tests and participation in clinical trials. Furthermore, patient autonomy is usually assumed to be assured by information. However, ensuring patient autonomy through informed consent is not an easy task [63] since individuals, in general, have great difficulties understanding genetic risk information [59, 65]. Furthermore, classical consent procedures follow with little time for the patient to be truly informed, and opt-out procedures may present genetic testing as a standard procedure. An offer to be genotyped from health care can be thereby be misinterpreted as a recommendation [66]. It has even been questioned whether it is ethical to assign patients the responsibility for medical decisions at all as they do not have the required medical education [64] and the information needed to make decisions involves too high levels of uncertainty [65, 67].

These difficulties raise the importance of not abandoning patients with information and choices, but rather inviting them in the deliberation process. It does not, however, imply that the choices should be taken away from patients. Just as patients are not obliged to make medical decisions they do not feel equipped to make, they have the right to make decisions about matters that affect their lives without fully knowing the consequences of their decision, from the perspective that it is the best choice for them and their life situation.

Several studies of this review found that many individuals have a positive attitude toward receiving genetic results [28, 29, 39, 40]. Communication, even if difficult, can with the help of patient’s preferences be built and contextualized to better fit their understanding [68], and should follow best practices as suggested by the scientific societies [69, 70]. One recommendation is that the return of genetic information should be followed by a process with an expert in the field and in many countries genetic counseling is required by law.

In precision medicine, the possibility to genetically profile an individual to enable a treatment choice is very different from genomic results in general and is linked to higher or lower chances for the treatment to work. While this may be a challenge, it is still the right of the patient to take a decision unless he or she specifically gives this up.

Several of the included studies of this review address the need to inform patients about data sharing to enable precision medicine. In a German study, physicians experienced that patients can be ignorant of the sensitivity of data, since they are in a desperate situation where they want help for an acute problem, and therefore are willing to sign (almost) anything. Data protection was therefore considered a special responsibility of physicians and researchers [63]. In another study, physicians in the US had the opposite experience and struggled with patients’ concerns about placing genetic information in their electronic health records, with fear that it would be shared with employers and insurance companies [59]. The different attitudes to sensitive data in these two studies might, to some extent, reflect the different health care systems, where health insurance is a much more crucial matter in the US compared with Germany and many other parts of Europe.

Other geographical differences might also influence how applicable the findings of this review are to a specific country or region. For instance, the European Society of Human Genetics (ESHG) recommends that genome analysis should be restricted to the original health problem. At the same time, other organizations have argued that 'actionable' genetic variants should or could be reported, e.g., the American College of Medical Genetics and Genomics, French Society of Predictive and Personalized Medicine, and Genomics England [66]. Hence, different clinical settings will manage different types and amounts of genetic results. Furthermore, media reporting and direct-to-consumer marketing influence public and patient perceptions and expectations and are likely to vary greatly between countries. The studies addressing direct-to-consumer marketing and media reporting in this review are mainly from North America, while direct-to-consumer marketing of prescribed pharmaceuticals is not allowed in many other countries. Differences based on culture and health care systems may decrease the generalizability of the findings of this study*.* Furthermore, our search did not identify many studies from low- and middle-income countries, indicating that research in these countries is needed.

The black-box problem (or the opacity problem as it is also referred to) of AI was considered a barrier to communication and was assumed to create issues regarding responsibility. However, there were no empirical examples from clinical oncology of this among the included papers. Future studies should therefore explore if these concerns are justified and, if they are, how they will unfold. That would be necessary to proceed from identifying opacity as an issue and actually manage the problem. The opacity of AI may evoke patients’ fears and concerns about its potential harm. Ploug & Holm (2020) argue that patients have the right to refuse AI involvement in decision-making, since acting on rational concern is to exercise rational autonomy and agency. Patients also have a formal right to refuse involvement of AI through the right to informed consent. For citizens of the European Union, the right “not to be subject to a decision based solely on automated processing” is guaranteed by article 22 of the General Data Protection Regulation (GDPR) if the decision significantly affects a person (Art. 22 GDPR 2018) [71].

Another finding of this review is the overly optimistic expectations of patients for precision medicine. AI and precision medicine are related to new technical innovations. The public has unconsciously associated success with new technological products with a “superiority” over existing technology [72]. Therefore, the overly optimistic expectations about precision medicine and AI may decrease as the “newness” fades. As time passes, and new therapies are developed, the implication of genetic information might also change character. For instance, being a carrier of a BRCA mutation no longer only implies being at high risk and having a hereditary form of cancer, but may also imply access to new targeted drugs [42].

This systematic review aimed to map out the consequences of precision medicine on the doctor-patient relationship. Epstein (2007) provides a framework for improving Patient-Centered Communication in Cancer Care that may be useful for improving areas where PM risks to jeopardize the doctor-patient relationship [8]. The framework addresses many of the challenges identified in this review, such as fostering a healthy relationship and managing uncertainty while also addressing skills training for health professionals. To manage the challenges connected to genetic testing, McGowan et al. (2014) suggest that providers draw lessons from the clinical genetics field when considering informed consent, privacy, and disclosure of results [59]. Since precision medicine entails technological complexity and scientific uncertainty, it may be even more important to emphasize the affective behaviors important for a good doctor-patient relationship. Thereby being open and honest about the uncertainty while expressing a willingness to act in the patient’s best interest [12, 13], while showing empathic understanding [12, 13]. Hunter states that there is an urgent need to develop methods to communicate uncertainty, help patients make sense of large amounts of complex information, and help them make choices among increasingly numerous options [73]. Furthermore, Hunter requests that these methods should help physicians answer a question valued by patients: “What would you do, doctor?” [73].

## Limitations

This systematic review has several limitations. Precision medicine involves different technologies. Hence the papers of this review address different aspects, such as the use of AI, molecular and genomic testing, data sharing. All distinct areas that could be analyzed separately. By looking at precision medicine in broad terms, it is possible that small but important issues related to either of these distinct areas have been underrepresented. Furthermore, the doctor-patient relationship is not clearly defined in the literature. Therefore, studies concerning this topic are not easily retrievable. Articles that do not fit our definitions may have been overlooked. The risk of this may be increased by the use of a qualitative narrative synthesis where the included studies were re-interpreted by the authors to answer the objective of this paper.

There was an overrepresentation of studies from the US and Canada, followed by a few European countries, while only two studies were conducted outside North America and Europe. The geographical context, including the health care system and culture of the included studies, should be considered before generalizing the findings. Furthermore, it is not certain that the results can be generalized to all cancer types, as perceptions may differ due to the severity and available treatment options, as well as the influence of family heredity for the specific cancer type.

## Conclusion

Many findings have previously been addressed in the field of doctor-patient communication and clinical genetics. Precision medicine adds complexity to these fields and further emphasizes the importance of clear communication. For instance, about the distinction between genomic and gene expression, and what patients can expect in terms of access, eligibility, effectiveness, and side-effects targeted therapies.

### Supplementary Information

**Additional file 1.**

**Additional file 2.**

## Data Availability

The data used during the current study are available from the corresponding author on reasonable request.

## Abbreviations

AI: Artificial intelligence
ESHG: European Society of Human Genetics
GDPR: General Data Protection Regulation
MMAT: The Mixed Methods Appraisal Tool
NSG: Next-generation sequencing
PRISMA: Preferred Reporting Items for Systematic Reviews and Meta-Analyses
SDM: Shared decision‐making
SPIDER criteria: Sample, phenomena of interest, design, evaluation, research type
VUS: Variants of uncertain significance
