# Supplementary material for: Precision cancer medicine and the doctor-patient relationship: a systematic review and narrative synthesis
Source: BMC Med Inform Decis Mak. 2023 Dec 14;23:286. doi: 10.1186/s12911-023-02395-x (PMC10722840; doi:10.1186/s12911-023-02395-x)
Supplement: Supplementary file 1 — Additional file 1. [file 12911_2023_2395_MOESM1_ESM.docx]

Supplementary file A. Search strategy and documentation.

Table 1. Search strategy and documentation **PubMed**, NCIB. **Date** 2021-12-06

| Search nr | Term | Results |
| --- | --- | --- |
| *Concept 1* | | |
| 1 | Professional-Patient Relations [MeSH] | 146,558 |
| 2 | doctor boundar* OR doctor communication* OR doctor interaction* OR doctor-patient communication* OR doctor-patient interaction* OR doctor-patient relation* OR obligation* OR patient autonom* OR patient boundar* OR patient communication* OR patient interaction* OR “patient value*” OR personal boundar* OR personal communication* OR personal interaction* OR physician boundar* OR physician communication* OR physician interaction OR physician-patient communication* OR physician-patient interaction* OR physician-patient relation* OR professional boundar* OR professional communication* OR professional interaction* OR professional-patient communication* OR professional-patient interaction* OR professional-patient relation* OR trust* [Title/Abstract] | 109,429 |
| 3 | 1 OR 2 | 236,748 |
| *Concept 2* | | |
| 4 | Algorithms OR Precision Medicine [MeSH] | 396,327 |
| 5 | AI OR algorithm* OR "artificial intelligence" OR "machine learning" OR "personalised care" OR "personalised cancer care" OR “personalised cancer medicine” OR “personalised medicine” OR “personalised prognos*” OR "personalized care" OR "personalized cancer care" OR "personalized cancer medicine" OR "personalized medicine" OR “personalized prognos*” OR "precision medicine" OR "precision oncolog*" [Title/Abstract] | 405,045 |
| 6 | 4 OR 5 | 638,599 |
| *Concept 3* | | |
| 7 | Neoplasms [MeSH] | 3,581,001 |
| 8 | cancer* OR neoplasm* OR oncolog* OR tumor* OR tumour* [Title/Abstract] | 3,279,262 |
| 9 | 7 OR 8 | 4,552,686 |
| *Final search* | 3 AND 6 AND 9 | 436 |

Table 2. Search strategy and documentation **Scopus,** Elsevier. **Date: 2021-12-07**

| Search nr | Term | Results |
| --- | --- | --- |
| *Concept 1* | | |
| 1 | doctor boundar* OR doctor communication* OR doctor interaction* OR doctor-patient communication* OR doctor-patient interaction* OR doctor-patient relation* OR obligation* OR patient autonom* OR patient boundar* OR patient communication* OR patient interaction* OR “patient value*” OR personal boundar* OR personal communication* OR personal interaction* OR physician boundar* OR physician communication* OR physician interaction OR physician-patient communication* OR physician-patient interaction* OR physician-patient relation* OR professional boundar* OR professional communication* OR professional interaction* OR professional-patient communication* OR professional-patient interaction* OR professional-patient relation* OR trust* [Title/abstract/keywords] | 798 |
| *Concept 2* | | |
| 2 | AI OR algorithm* OR "artificial intelligence" OR "machine learning" OR "personalised care" OR "personalised cancer care" OR “personalised cancer medicine” OR “personalised medicine” OR “personalised prognos*” OR "personalized care" OR "personalized cancer care" OR "personalized cancer medicine" OR "personalized medicine" OR “personalized prognos*” OR "precision medicine" OR "precision oncolog*" [Title/abstract/keywords] | 3,937,697 |
| *Concept 3* | | |
| 3 | cancer* OR neoplasm* OR oncolog* OR tumor* OR tumour* [Title/Abstract/keywords] | 5,421,169 |
| *Final search* | 1 AND 2 AND 3 | 0 |

Table 3. Search strategy and documentation **Web of Science** **Core Collection**, Clarivate. **Date: 2021-12-07**

| Search nr | Term | Results |
| --- | --- | --- |
| *Concept 1* | | |
| 1 | doctor boundar* OR doctor communication* OR doctor interaction* OR doctor-patient communication* OR doctor-patient interaction* OR doctor-patient relation* OR obligation* OR patient autonom* OR patient boundar* OR patient communication* OR patient interaction* OR “patient value*” OR personal boundar* OR personal communication* OR personal interaction* OR physician boundar* OR physician communication* OR physician interaction OR physician-patient communication* OR physician-patient interaction* OR physician-patient relation* OR professional boundar* OR professional communication* OR professional interaction* OR professional-patient communication* OR professional-patient interaction* OR professional-patient relation* OR trust* [Topic] | 654,193 |
| *Concept 2* | | |
| 2 | AI OR algorithm* OR "artificial intelligence" OR "machine learning" OR "personalised care" OR "personalised cancer care" OR “personalised cancer medicine” OR “personalised medicine” OR “personalised prognos*” OR "personalized care" OR "personalized cancer care" OR "personalized cancer medicine" OR "personalized medicine" OR “personalized prognos*” OR "precision medicine" OR "precision oncolog*" [Topic] | 2,533,060 |
| *Concept 3* | | |
| 3 | cancer* OR neoplasm* OR oncolog* OR tumor* OR tumour* [Topic] |  |
| *Final search* | 1 AND 2 AND 3 | 2,971 |

Table 4. Search strategy and documentation Social Science Premium Collection, Proquest. **Date: 2021-12-08**

| Search nr | Term | Results |
| --- | --- | --- |
| *Concept 1* | | |
| 1 | MAINSUBJECT.EXACT("Professional relationships") | 9,943 |
| 2 | doctor boundar* OR doctor communication* OR doctor interaction* OR doctor-patient communication* OR doctor-patient interaction* OR doctor-patient relation* OR obligation* OR patient autonom* OR patient boundar* OR patient communication* OR patient interaction* OR “patient value*” OR personal boundar* OR personal communication* OR personal interaction* OR physician boundar* OR physician communication* OR physician interaction OR physician-patient communication* OR physician-patient interaction* OR physician-patient relation* OR professional boundar* OR professional communication* OR professional interaction* OR professional-patient communication* OR professional-patient interaction* OR professional-patient relation* OR trust* (Document title) | 71,995 |
| 3 | doctor boundar* OR doctor communication* OR doctor interaction* OR doctor-patient communication* OR doctor-patient interaction* OR doctor-patient relation* OR obligation* OR patient autonom* OR patient boundar* OR patient communication* OR patient interaction* OR “patient value*” OR personal boundar* OR personal communication* OR personal interaction* OR physician boundar* OR physician communication* OR physician interaction OR physician-patient communication* OR physician-patient interaction* OR physician-patient relation* OR professional boundar* OR professional communication* OR professional interaction* OR professional-patient communication* OR professional-patient interaction* OR professional-patient relation* OR trust* (Abstract) | 95,297 |
| 4 | 1 OR 2 OR 3 | 170,328 |
| *Concept 2* | | |
| 5 | MAINSUBJECT.EXACT("Algorithms") OR MAINSUBJECT.EXACT("Artificial intelligence") OR MAINSUBJECT.EXACT("Precision medicine") | 56,194 |
| 6 | AI OR algorithm* OR "artificial intelligence" OR "machine learning" OR "personalised care" OR "personalised cancer care" OR “personalised cancer medicine” OR “personalised medicine” OR “personalised prognos*” OR "personalized care" OR  "personalized cancer care" OR "personalized cancer medicine" OR "personalized medicine" OR “personalized prognos*” OR "precision medicine" OR "precision oncolog*" (Document title) | 21,939 |
| 7 | AI OR algorithm* OR "artificial intelligence" OR "machine learning" OR "personalised care" OR "personalised cancer care" OR “personalised cancer medicine” OR “personalised medicine” OR “personalised prognos*” OR "personalized care" OR  "personalized cancer care" OR "personalized cancer medicine" OR "personalized medicine" OR “personalized prognos*” OR "precision medicine" OR "precision oncolog*" (Abstract) | 75,053 |
| 8 | 5 OR 6 OR 7 | 103,313 |
| *Concept 3* | | |
| 9 | MAINSUBJECT.EXACT("Cancer") OR MAINSUBJECT.EXACT("Oncology") OR MAINSUBJECT.EXACT("Tumors") | 64,110 |
| 10 | cancer* OR neoplasm* OR oncolog* OR tumor* OR tumour* (Document title) | 66,574 |
| 11 | cancer* OR neoplasm* OR oncolog* OR tumor* OR tumour* (Abstract) | 100,455 |
| 12 | 9 OR 10 OR 11 | 131,030 |
| *Final search* | 4 AND 8 AND 12 | 30 |
