# Supplementary material for: Precision cancer medicine and the doctor-patient relationship: a systematic review and narrative synthesis
Source: BMC Med Inform Decis Mak. 2023 Dec 14;23:286. doi: 10.1186/s12911-023-02395-x (PMC10722840; doi:10.1186/s12911-023-02395-x)
Supplement: Supplementary file 2 — Additional file 2. [file 12911_2023_2395_MOESM2_ESM.docx]

**Supplementary file B. Critical appraisal of the methodological quality of included studies conducted using the latest version of the mixed methods appraisal tool (MMAT)**, **version 2018**

Note: all studies answered ‘yes’ to the first two screening questions of the MMAT:

S.1. Are there clear research questions?

S.2. Do the collected data allow to address the research questions?

*mixed methods studies. Following the instruction of the MMAT guidance the mixed-methods studies first were assessed on their qualitative and quantitative components independently, and finally using the questions 5.1. – 5.5 on their mixed-methods methodology.

| 1. Qualitative studies | | | | | | |
| --- | --- | --- | --- | --- | --- | --- |
| First author | Year | Q1.1 | Q1.2 | Q1.3 | Q1.4 | Q1.5 |
| Abe | 2017 | Yes | Yes | Yes | Yes | Yes |
| Best* | 2020 | Yes | Yes | Yes | Yes | Yes |
| Bijlsma | 2018 | Yes | Yes | Yes | Yes | Yes |
| Bombard | 2014 | Yes | Yes | Yes | Yes | Yes |
| Bombard | 2015 | Yes | Yes | Yes | Yes | Yes |
| Costa | 2021 | Yes | Yes | Yes | Yes | Yes |
| Dodson | 2017 | Yes | Can’t tell | Can’t tell | No | Can’t tell |
| Hamilton | 2017 | Yes | Yes | Yes | Yes | Yes |
| Hamilton | 2021 | Yes | Yes | Yes | Yes | Yes |
| Harris | 2013 | Yes | Yes | Yes | Yes | Yes |
| Kerr | 2019 | Yes | Yes | Yes | Yes | Yes |
| Mamzer | 2017 |  |  |  |  |  |
| McCradden | 2020 | Yes | Yes | Yes | Yes | Yes |
| Perry | 2017 | Yes | Yes | Yes | Yes | Yes |
| Pellegrini | 2011 | Yes | Yes | Yes | Yes | Yes |
| Rattay | 2018 | Yes | Yes | Yes | Yes | Yes |
| Pichler | 2020 | Yes | Yes | Yes | Yes | Yes |
| Rohrmoser | 2019 | Yes | Yes | Yes | Yes | Yes |
| Steltzer | 2020 | Yes | Yes | Can’t tell | Can’t tell | Can’t tell |
| Therond | 2020 | Yes | Yes | Yes | Yes | Yes |
| Wright | 2019 | Yes | Yes | Yes | Yes | Yes |
| Q1.1. Is the qualitative approach appropriate to answer the research question?  Q1.2. Are the qualitative data collection methods adequate to address the research question?  Q1.3. Are the findings adequately derived from the data?  Q1.4. Is the interpretation of results sufficiently substantiated by data?  Q 1.5. Is there coherence between qualitative data sources, collection, analysis and interpretation? | | | | | | |

| 4. Quantitative descriptive studies | | | | | | |
| --- | --- | --- | --- | --- | --- | --- |
| First author | Year | Q4.1 | Q4.2 | Q4.3 | Q4.4 | Q4.5 |
| Best* | 2020 | Yes | Yes | Yes | Yes | Yes |
| Issa | 2013 | Yes | Yes | Can’t tell | Can’t tell | Can’t tell |
| Soellner | 2021 | Can’t tell | Can’t tell | Can’t tell | Yes | Yes |
| Yang | 2019 | Yes | Yes | Yes | Yes | Yes |
| Q 4.1. Is the sampling strategy relevant to address the research question?  Q 4.2. Is the sample representative of the target population?  Q 4.3. Are the measurements appropriate?  Q 4.4. Is the risk of nonresponse bias low?  Q 4.5. Is the statistical analysis appropriate to answer the research question? | | | | | | |

| 5. Mixed methods studies | | | | | | |
| --- | --- | --- | --- | --- | --- | --- |
| First author | Year | Q5.1 | Q5.2 | Q5.3 | Q5.4 | Q5.5 |
| Best* | 2020 | Yes | Yes | Yes | Yes | Yes |
| Q 5.1. Is there an adequate rationale for using a mixed methods design to address the research question?  Q 5.2. Is the integration of qualitative and quantitative data relevant to address the research question?  Q 5.3. Are the outputs of the integration of qualitative and quantitative components adequately interpreted?  Q 5.4. Are divergences and inconsistencies between quantitative and qualitative results adequately addressed?  Q 5.5. Do the different components of the study adhere to the quality criteria of each tradition of the methods involved? | | | | | | |
